# Multi-decadal high-resolution historical simulations with the ECMWF global atmosphere model

**DOI:** 10.1038/s41597-026-07388-9

**Published:** 2026-08-18

**Authors:** Matthias Aengenheyster, Christopher David Roberts, Razvan Aguridan, Matthew Griffith, Robin Hogan, Balthasar Reuter, Malcolm J Roberts, Domokos Sarmany, Retish Senan, Timothy N. Stockdale, Sebastien Villaume, Fabian Wachsmann

**Affiliations:** 1 ECMWF, Research Department, Bonn, Germany; 2 ECMWF, Research Department, Reading, UK; 3 formerly ECMWF, Forecast and Services Department, Bonn, Germany; 4 ECMWF, Forecast and Services Department, Reading, UK; 5 Met Office, Hadley Centre, Exeter, UK; 6 German Climate Computing Centre, Data Management, Hamburg, Germany

**Keywords:** Atmospheric dynamics, Climate and Earth system modelling

## Abstract

This paper describes an ensemble of global atmosphere reference simulations covering the period 1980 to 2023 produced with the European Centre for Medium-Range Weather Forecasts (ECMWF) Integrated Forecasting System (IFS). The resulting dataset consists of 6-hourly three-dimensional global outputs from higher- and lower-resolution configurations of the IFS, which have average horizontal grid spacings of ~9 km (one ensemble member) and ~28 km (ten ensemble members), respectively. The atmosphere is constrained by high-resolution satellite-based estimates of daily mean sea surface temperature and sea ice concentration and time-evolving external climate forcings, including observed greenhouse gas and estimated aerosol concentrations. These data were produced as part of the European Eddy-Rich Earth System Models (EERIE) project and will serve as a reference for corresponding ocean-atmosphere coupled simulations and idealised sensitivity experiments. We have made this dataset publicly available and expect it to be a valuable resource for scientific research and other applications that extend beyond the lifetime of the EERIE project.

## Background & Summary

The European Centre for Medium-Range Weather Forecasts (ECMWF) Integrated Forecasting System (IFS) is a global coupled Earth System Model (ESM), which includes dynamic representations of the atmosphere, ocean, land surface, and ocean waves^1^. The IFS is used operationally at ECMWF to produce medium-range (up to 15 days) and extended-range (up to 46 days) weather forecasts and seasonal outlooks of ocean and atmosphere conditions up to 13 months into the future.

The IFS can also be run in climate model configurations^2–4^ and for many years has been a core component of the EC-Earth climate model^5,6^. One of the major differences between weather and climate configurations of the IFS is the horizontal resolution of the atmosphere, which has typically been much coarser in climate contexts. For example, IFS-based simulations contributing to the High-Resolution Model Intercomparison Project (HighResMIP) and phase 6 of the Coupled Model Intercomparison Project (CMIP6) have a minimum horizontal grid-spacing of 28 km in the atmosphere^7^. In contrast, the operational implementation of IFS Cycle 48r1 on 27 June 2023 boosted the horizontal resolution of medium-range ensemble forecasts to an average grid horizontal spacing of ~9 km. Here, we present data from free-running atmosphere-only climate simulations covering the period 1980 to 2023 produced with two configurations of the IFS derived from Cycle 48r1:

- Ten members from IFS_48R1_Tco399_EERIE, which has an average horizontal grid spacing of ~28 km, which matches the highest resolution atmosphere simulations that contributed to HighResMIP/CMIP6. Sometimes called Tco399 for brevity.
- One member from IFS_48R1_Tco1279_EERIE, which has an average horizontal grid spacing of ~9 km, which matches the resolution of ECWMF operational medium-range weather forecasts. Sometimes called Tco1279 for brevity.

**Fig. 1:**
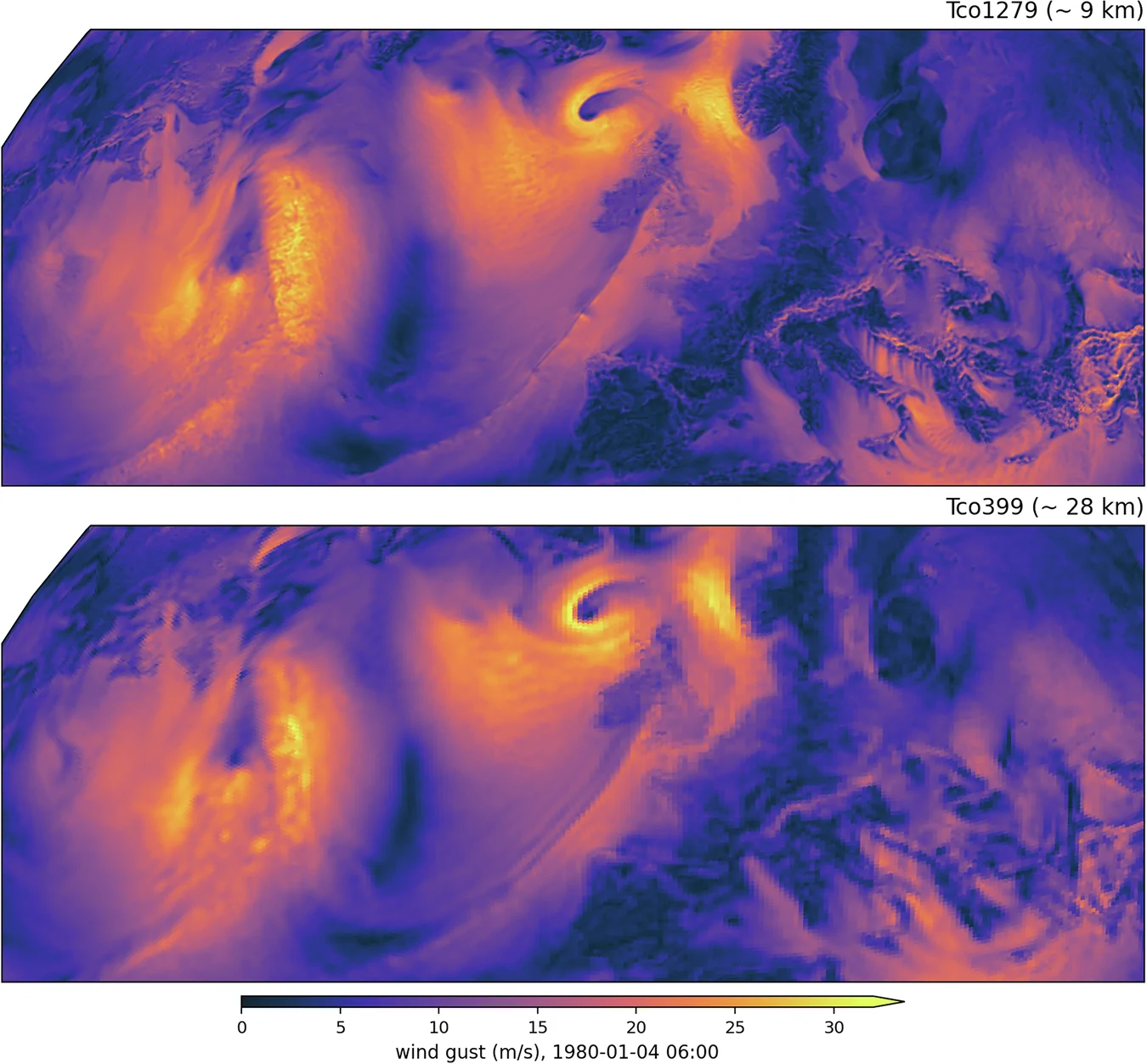
Instantaneous wind gust speed four days after initialization in (top) IFS_48R1_Tco1279_EERIE^40^ and (bottom) the first member of IFS_48R1_Tco399_EERIE^41^, plotted on the native model grid. The large-scale atmospheric circulation features are very similar due to the shared ERA5 initial conditions and short lead time. However, IFS_48R1_Tco1279_EERIE has a much higher horizontal resolution that better resolves small-scale atmospheric processes, including sharp gradients in wind gust speed.

The impact of these differences in horizontal resolution on atmospheric processes is illustrated in Fig. 1. We follow previous studies^2^ and use the term ‘atmosphere-only’ to identify model configurations that do not include interactive ocean or sea ice models, but retain two-way coupling with interactive land-surface and ocean-wave models. Each simulation is constrained by time-evolving external climate forcings, including observed greenhouse gas and aerosol concentrations, and high-resolution satellite-based estimates of daily mean sea surface temperature and daily mean sea ice concentration.

The resulting dataset, consisting of comprehensive 6-hourly three-dimensional global outputs, was produced as part of the European Eddy-Rich Earth System Models (EERIE) project^8^ and will be used in combination with corresponding coupled simulations and other idealised atmosphere-only experiments to provide insights into the impacts of ocean coupling, atmospheric resolution, and the role of the ocean mesoscale for weather and climate variability. A preliminary description of an incomplete version of this dataset can be found in the associated EERIE project deliverable report^9^, elements of which are reproduced and extended here. These atmosphere-only experiments also share an atmospheric configuration with coupled experiments run as part of the Destination Earth^10^ Initiative Climate Adaptation Twin (climateDT) and the nextGEMS^11^ project. We expect these data to have scientific value as part of multi-model datasets (e.g. CMIP^12^) or for analysis of systematic errors and their resolution-sensitivity in a free-running version of the ECMWF atmosphere model without the influence of data assimilation or forecast initialisation. Another possible use of this data is for training deep-learning models of the Earth’s atmosphere, which have previously used data produced by HighResMIP/CMIP6^13,14^.

## Methods

### The ECWMF Integrated Forecasting System

The atmosphere-only simulations described in this study are based on IFS Cycle 48r1^1^ which was operational between 27 June 2023 and 12 November 2024. We adopt several modifications to the operational implementation developed as part of the NextGEMS project^3,4^. These include the activation of tracer mass fixers to improve global energy/moisture conservation and parameter tuning to improve the top-of-atmosphere (TOA) radiation balance in higher resolution configurations. One deviation from the NextGEMS configuration^4^ is that we do not reduce the activity of the parametrised cloud-base mass flux, which has been shown to be beneficial in IFS simulations with grid spacing of ~4 km^15^. Instead, we adopt the standard operational settings for the deep convection scheme, which provides superior performance for the atmospheric resolutions considered here.

The atmospheric component of the IFS is based on a two-time-level semi-implicit, semi-Lagrangian dynamical core with computations alternated between spectral and cubic octahedral reduced Gaussian (Tco) grid-point representations each time step^16–21^. We use a time step of 450 seconds with the Tco1279 grid (~9 km grid spacing) and a time step of 900 seconds with the Tco399 grid (~28 km grid spacing). Both configurations use 137 vertical levels and, for computational efficiency, use 32-bit ‘single‘ precision for (almost) all floating point calculations^22,23^. We do not use any stochastic perturbations to parameterised model physics. Ensembles are generated by perturbing ERA5^24^ atmosphere initial conditions for January 1^st^ following the same process that is applied in operational ECMWF ensemble (re)forecasts^25,26^ using perturbations generated from a combination of singular vectors^27^ and the ERA5 ensemble of data assimilations (EDA). The specific code versions for the IFS and associated software stack used to produce these experiments are provided in Table 1. The atmosphere is coupled to the land surface model ECLand^28^ with four soil levels, and the ocean wave model ecWAM^29^.

**Table 1:** Tag names and version numbers of important IFS components.

ifs-bundle	DE_CY48R1.0_EERIE_20240726
ifs-source	DE_CY48R1.0_climateDT_20240718
RAPS	0.1.9
multio	2.1.9
eccodes	2.36.1
fdb5	5.12.0
MIR	1.22.0

### Time-varying model boundary conditions

The simulations follow a revised version of the CMIP6/HighResMIP protocols^6,30^, which are proposed for HighResMIP2^31^. External climate forcings in the IFS are mostly specified as described in Roberts *et al*.^2^, with some modifications. In particular, tropospheric aerosols in IFS simulations are specified as time-evolving climatologies generated using methods developed within the CONsistent representation of temporal variations of boundary Forcings in reanalysES and Seasonal forecasts (CONFESS) project^32,33^. These climatologies are derived from IFS simulations with Cy48r1 prognostic atmospheric chemistry driven by revised versions of CMIP6 emissions data, in particular CEDS v_2021-4-21 anthropogenic emissions and GFED version 4s fire emissions. The atmospheric circulation in these simulations is constrained to track the observed meteorological conditions as represented by the ERA5 reanalysis, resulting in an emissions-driven pseudo-reanalysis of the atmospheric chemistry and aerosols. The climatology of each aerosol species is created by linear interpolation between 9-year running means centred at 3-year intervals from 1975 to 2015. For dates after 2015, a fixed 9-year aerosol climatology for 2011–2019 is used, so that recent changes in tropospheric aerosols are not represented.

From 1980–2014, greenhouse gases (i.e. CO_2_, CH_4_, N_2_O, CFC11 and CFC12), volcanic aerosols, ozone, and total solar irradiance are specified to follow CMIP6 historical data as described in previous work^2^. For the period after 2015, for which CMIP6 historical data are not available, we specify external forcings as follows: (i) greenhouse gases and ozone are specified to follow the SSP3-7.0 scenario, (ii) volcanic aerosol optical depths are held constant using a representative “no major emissions” year, and (iii) solar forcings are specified using the CMIP6 reference estimate of most likely solar activity from 2015 to 2300. Other external boundary conditions, including land surface properties, are specified as seasonally varying climatologies that do not evolve with time.

**Fig. 2:**
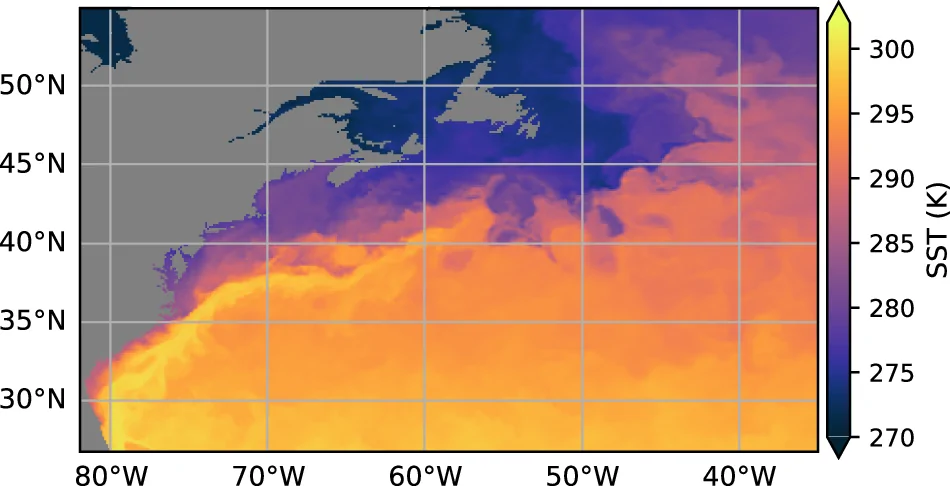
Daily mean sea surface temperature from ESA CCI SST v3.0.1^34^ in the Gulf Stream region for January 1st 2020.

Time-evolving ocean and sea ice boundary conditions in these atmosphere-only simulations are prescribed using high-resolution satellite-based estimates of sea surface temperature (SST) and sea ice concentration (SIC). SSTs are specified using data from the European Space Agency Climate Change Initiative Sea Surface Temperature (ESA CCI SST version 3.0.1), which provides daily mean ocean temperatures representative of 20 cm depth from 1980 to present^34^. Daily mean sea ice concentrations are specified using data from European Organisation for the Exploitation of Meteorological Satellites (EUMETSAT) Ocean and Sea Ice Satellite Application Facility (OSI-SAF). Specifically, we use the OSI-450 and OSI-430-b sea ice concentration products that are distributed alongside - and used as inputs for - the ESA CCI SST v3.0.1 analysis^34^. Importantly, the ESA CCI SST data have sufficient horizontal resolution to resolve sharp gradients associated with ocean fronts and mesoscale ocean eddies (Fig. 2). SST and sea ice data were retrieved on the original 0.05^°^ × 0.05^°^ latitude-longitude grid and then conservatively interpolated to the required cubic octahedral reduced Gaussian grids using the MIR software^35^.

Compared to previous versions, ESA CCI SST v3.0.1 has improved accuracy and temporal consistency, especially during the 1980s and early 1990s^34^. For example, ESA CCI SST v3.0.1 does not exhibit spurious variability of daily mean global SST anomalies during the 1980s and early 1990s, which are evident in other SST data sets (not shown). Despite these improvements to data consistency, there are some characteristics of ESA CCI SST v3.0.1 that evolve with time. In particular, although the SST data is distributed on a 0.05^°^ × 0.05^°^ grid, the feature resolution is typically larger than 15 km and is generally coarser during the earlier period due to lack of high-resolution satellite sensors^34^.

### High Performance Computing

IFS simulations were run on the ECMWF Atos Sequana XH2000 supercomputer, where all outputs were written to disk within a Fields DataBase (FDB)^36–38^. Each ensemble member was run as a parallel job on either 80 compute nodes for Tco1279 or 8 nodes for Tco399, with 128 cores per node. The average throughput was 1.28 simulated years per day (SYPD) for Tco1279 and 2.77 SYPD for Tco399. The impact of batch job queuing reduced effective throughput to 1.15 SYPD for IFS Tco1279 simulations but was negligible for IFS Tco399.

IFS model output and in-memory data processing was handled by MultIO^39^, a software package that provides I/O-server functionality for distributed Earth System Models and user-configurable post-processing pipelines, including online calculation of temporal averages and interpolation to non-native grids. Fluxes were accumulated online from model step data and thus derived average rates are exact. Daily and monthly means for other fields were computed online in the MultIO pipelines from hourly native resolution data for consistency with outputs produced in Destination Earth^10^ and nextGEMS^11^ projects. Importantly, this approach to the calculation of time averages deviates from the current practice for operational ECMWF seasonal and sub-seasonal forecasts, which derive weekly and monthly means from 6-hourly output.

All data were saved on both the native atmosphere model grid (either Tco1279 or Tco399) and a regular 0.25^°^ × 0.25^°^ latitude-longitude grid, to which they were re-gridded with a conservative interpolation method in the MultIO pipelines using MIR^35^. Each simulated model day produced either 11 GB (Tco1279) or 1.3 GB (Tco399) of native resolution output and 1.8 GB of output on a regular 0.25^°^ × 0.25^°^ latitude-longitude grid for a total data volume of ~0.7 PB.

## Data Records

All 6-hourly and daily mean output on the native model grid has been archived in the ECMWF operational Meteorological Archival and Retrieval System (MARS) under the new EERIE data class (“ed”) as data records IFS_48R1_Tco1279_EERIE^40^ for the Tco1279 single-member simulation and as IFS_48R1_Tco399_EERIE^41^ for the Tco399 10-member ensemble. The simulations have been included in the list of public research experiments (https://apps.ecmwf.int/ifs-experiments/).

To download the datasets, the ECMWF Web API python client is required. Please start from the ECMWF Web API documentation^42^ which includes links to install the python package ecmwf-api-client. It is recommended (but not required) to create a free ECMWF user account and configure the API key into the .ecmwfapirc file. The ecmwf-api-client executes MARS requests to download the data. In addition to retrievals at native model resolutions the client supports on-the-fly interpolation to a wide variety of horizontal grids (see examples and documentation). For convenience, the data records include sample code that span the range of available variables. These are meant to be modified according to user needs, noting that the examples retrieve very large amounts of data.

The output variables are listed in Table 2, separated into three groups. First, “Surface/single-level fields” are state variables with no vertical dimension, these can represent the Earth’s surface, the top of the atmosphere or vertical integrals. These were written as 6-hourly instantaneous fields, daily means and monthly means. Second, “Accumulated/flux fields” are fluxes such as radiation or precipitation. They also have no vertical dimension and were written as 6-hourly accumulations, daily mean rates and monthly mean rates. Third, “Pressure level fields” are three-dimensional atmospheric state variables on 19 vertical pressure levels. Like the first group these were written as 6-hourly instantaneous fields, daily means and monthly means.

**Table 2:** Available output variables from IFS atmosphere-only simulations.

Surface/single-level fields	Accumulated/flux fields
2 metre temperature (167, 228004)	Total precipitation (228, 172228)
2 metre dewpoint temperature (168, 235168)	Convective precipitation (143, 172143)
2 metre specific humidity (174096, 235089)	Snowfall (144, 172144)
10 metre wind speed (207, 228005)	Evaporation (182, 172182)
10 metre zonal wind (165, 235165)	Eastward surface stress (180, 235041)
10 metre meridional wind (166, 235166)	Northward surface stress (181, 235042)
Instantaneous 10 metre wind gust (228029, –)	Runoff (205, 172205)
Boundary layer height (159, 235159)	Surface runoff (8, 172008)
Sea ice area fraction (31, 235083)	Sub-surface runoff (9, 172009)
Sea surface temperature (34, 235084)	Latent heat flux (147, 235034)
Mean sea level pressure (151, 235151)	Sensible heat flux (146, 235033)
Snow depth (141, 235141)	Surface net shortwave radiation (176, 235037
Skin temperature (235, 235079)	Surface net shortwave radiation, clear-sky (210, 235051)
Surface pressure (134, 235134)	Surface net long-wave radiation (177, 235038)
Soil moisture on 4 levels (260199, 235077)	Surface net long-wave radiation, clear-sky (211, 235052)
Soil temperature on 4 levels (260360, 235094)	Surface short-wave radiation downwards (169, 235035)
Maximum 10 metre wind gust (–, 49)	Surface long-wave radiation downwards (175, 235036)
Maximum 2 metre temperature (51, 201)	Top incident short-wave radiation (212, 235053)
Minimum 2 metre temperature (52, 202)	Top net short-wave radiation (178, 235039)
Low cloud cover (186, 235186)	Top net short-wave radiation, clear sky (208, 235049)
Medium cloud cover (187, 235187)	Top net long-wave radiation (179, 235040)
High cloud cover (188, 235188)	Top net long-wave radiation, clear sky (209, 235050)
Total cloud cover (164, 228006)	
Total column cloud ice water (79, 235088)	**Pressure level fields**
Total column cloud liquid water (78, 235087)	Geopotential (129, 235129)
Total column water (136, 235136)	Temperature (130, 235130)
Total column vertically-integrated water vapour (137, 235137)	Zonal wind (131, 235131)
Significant wave height (140229, 141229)	Meridional wind (132, 235132)
Mean zero-crossing wave period (140221, 141221)	Vertical velocity (135, 235135)
Mean wave direction (140230, –)	Relative humidity (157, 235157)
Mean wave period (140232, 141232)	Specific humidity (133, 235133)
Peak wave period (140231, 141231)	

All variables are written as 6-hourly instantaneous values (accumulated for fluxes), and as daily and monthly means. For each descriptive name, the GRIB paramID is provided in parentheses first for the instantaneous / accumulated variable, and second the time-mean encoding. The latter is used for daily and monthly means. The descriptive name points to instantaneous / accumulated variable and is found in the file metadata under the ‘name’ key. Descriptive names differ for time-mean encodings. Time-means of accumulated variables are written as mean rates with corresponding change in unit. 3D fields are available on the following pressure levels: 1000, 925, 850, 700, 600, 500, 400, 300, 250, 200, 150, 100, 70, 50, 20, 10, 30, 5, 1 hPa. Maximum and minimum statistics are written as daily and monthly statistics. Mean wave direction is not written as a time-mean. Data requests from^40,41^ require specifying the paramID. Additional information can be found by searching the parameter database^45^ for paramIDs or descriptive names.

The data is written under GRIB2 standards^43^ as encoded by ecCodes^44^ 2.36.1. Each output variable is uniquely defined by its numeric paramId which is given in Table 2, which in the data request code is called “param”. Typically, for each physical variable in Table 2, two paramId are provided, these represent first the instantaneous variable and second the time-mean variable. Further documentation is available in the ECMWF parameter database^45^.

Both the 6-hourly and daily mean data is archived in the “clte” stream, distinguished by the two paramId for each physical variable. Monthly mean data will be archived under the “clmn” stream in the future using the time-mean paramId. Until then, monthly means can be constructed up to machine precision from the daily means, and additionally monthly means are hosted on DKRZ (see below).

Note that the shortName, often used to label variables in climate datasets, may vary depending on the eccodes version used to create or access the data. The variables, frequencies, and resolutions of output data reflect a balance between outputs required for comprehensive process-based evaluation of weather and climate variability, and efficient use of HPC resources and storage.

In addition to the MARS archive, a subset of the model output is freely available through the ‘EERIE cloud’^46,47^ hosted at the German Climate Computing Centre (DKRZ). The DKRZ-hosted datasets can be explored through a STAC-based data browser^48^, and also visualised interactively. In the EERIE cloud, IFS_48R1_Tco1279_EERIE is labelled “IFS-AMIP-TCO1279”^49^ and IFS_48R1_Tco399_EERIE is labelled “IFS-AMIP-TCO399”^50^. Primarily, the available data is on a regular 0.25^°^ × 0.25^°^ latitude-longitude grid, and includes the full set of monthly mean fields, as well as some daily mean and 6-hourly data. Sample python code is provided to directly stream the data to any internet-connected device. Usage of the DKRZ-hosted datasets is shown in the EERIE repositories on GitHub^51^.

## Technical validation

**Fig. 3:**
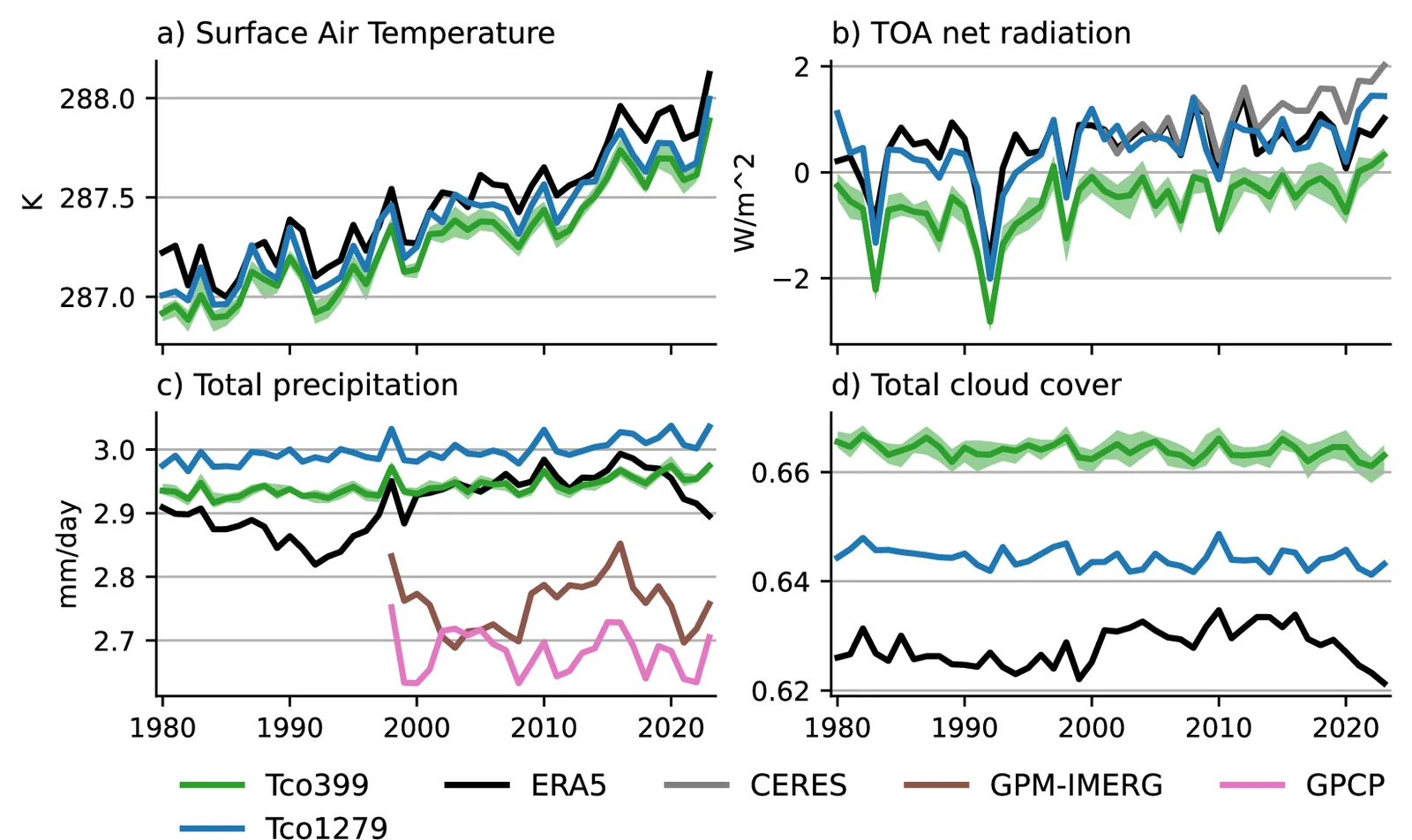
**(a)** Global mean 2 metre temperature, **(b)** global mean top-of-atmosphere net radiation (positive downwards), **(c)** global mean precipitation, and **(d)** global mean fractional cloud cover. Time series are plotted as annual means and are compared to equivalent data from ERA5^24^ (black), GPM-IMERG^55^ (brown), GPCP^54^ (pink) and CERES^52^ (grey).

This section provides a brief overview of the climate quality of IFS_48R1_Tco1279_EERIE^40^ and IFS_48R1_Tco399_EERIE^41^ atmosphere-only simulations, including evaluation of (1) global mean trends, (2) planetary energy budgets, and (3) mean biases. At both resolutions the IFS accurately captures long-term trends and interannual variability in global mean 2 metre temperature and net TOA radiation balance (Fig. 3) as represented in ERA5, which partly reflects the strong constraint from the specified SST boundary conditions. However, neither IFS configuration (nor ERA5) captures the recent increases Earth’s TOA energy imbalance as quantified by satellite-based observations^52^. This may be a consequence of common deficiencies in the prescribed aerosol forcings^53^.

One notable deficiency in the IFS Tco399 simulations is a ~1.5 Wm^−2^ negative bias in global mean net TOA radiation balance. In contrast, global mean net TOA radiation fluxes in Tco1279 simulations are in good agreement with mean estimates from the ERA5 reanalysis^24^ and closer to CERES observations^52^. These differences in planetary energy balance are a consequence of differences in cloud properties, which are sensitive to horizontal resolution (Fig. 3). The good agreement at Tco1279 is a consequence of the previous tuning of cloud-related parameters to improve the agreement with observations at higher resolutions^3,4^. For consistency, we have retained the same cloud parameters at both resolutions, despite the resulting negative bias in global mean net TOA radiation balance in the Tco399 configuration. This global mean bias has a very limited impact in the lower-resolution IFS atmosphere-only simulations due to the strong constraint from the imposed SSTs. However, we note that a similar magnitude bias could negatively impact coupled ocean-atmosphere climate simulations. For this reason, we would not recommend running multi-decadal coupled simulations with the Tco399 configuration of the IFS presented here without retuning cloud-related properties for improved TOA energy balance.

**Fig. 4:**
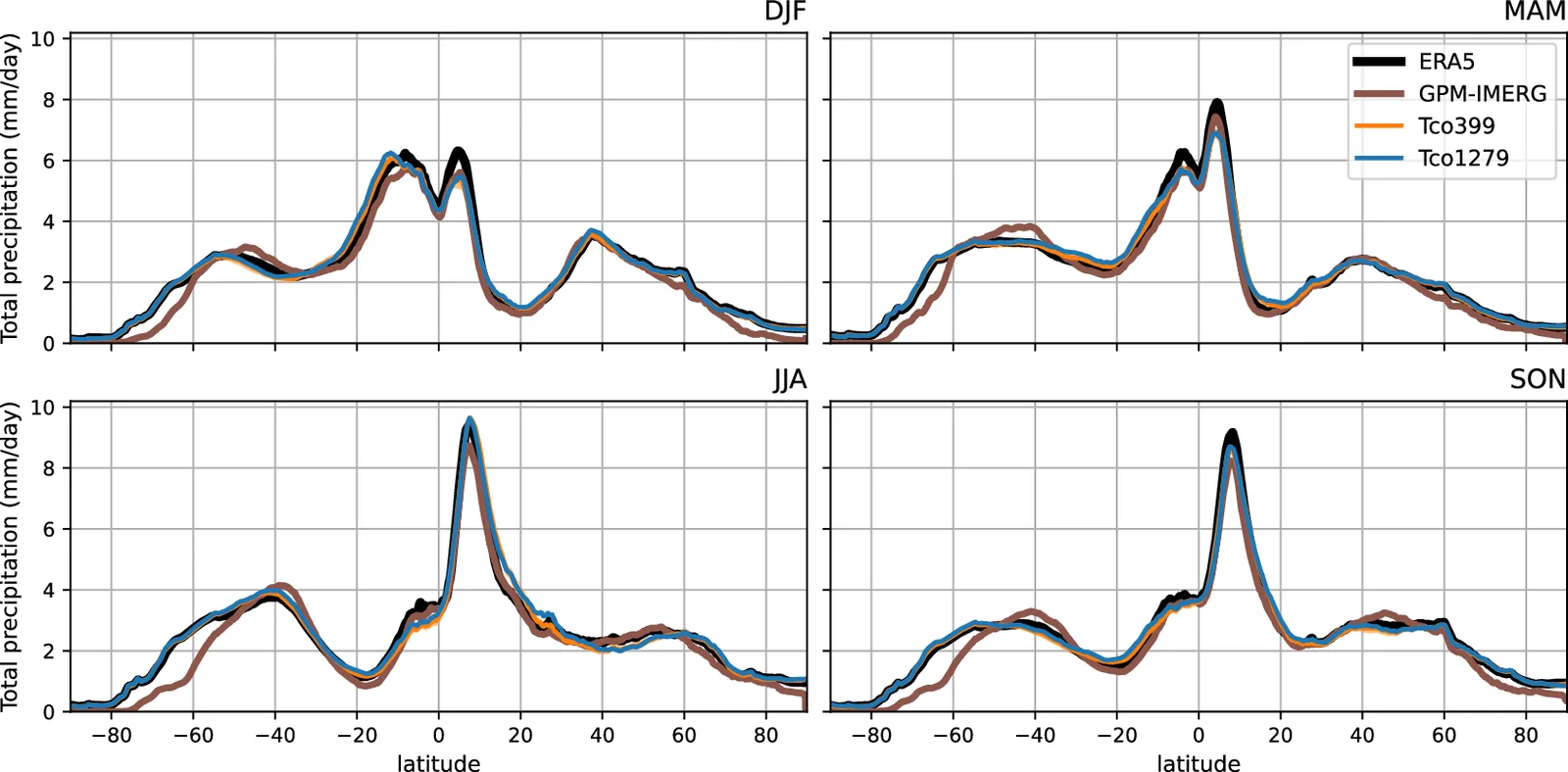
**(a)** Zonal mean total precipitation climatologies for the December-January-February (DJF) season calculated using data for the period 2010-2020. **(b**,**c**) As **(a)**, but for the March-April-May (MAM), June-July-August (JJA), and September-October-November (SON) seasons, respectively.

**Fig. 5:**
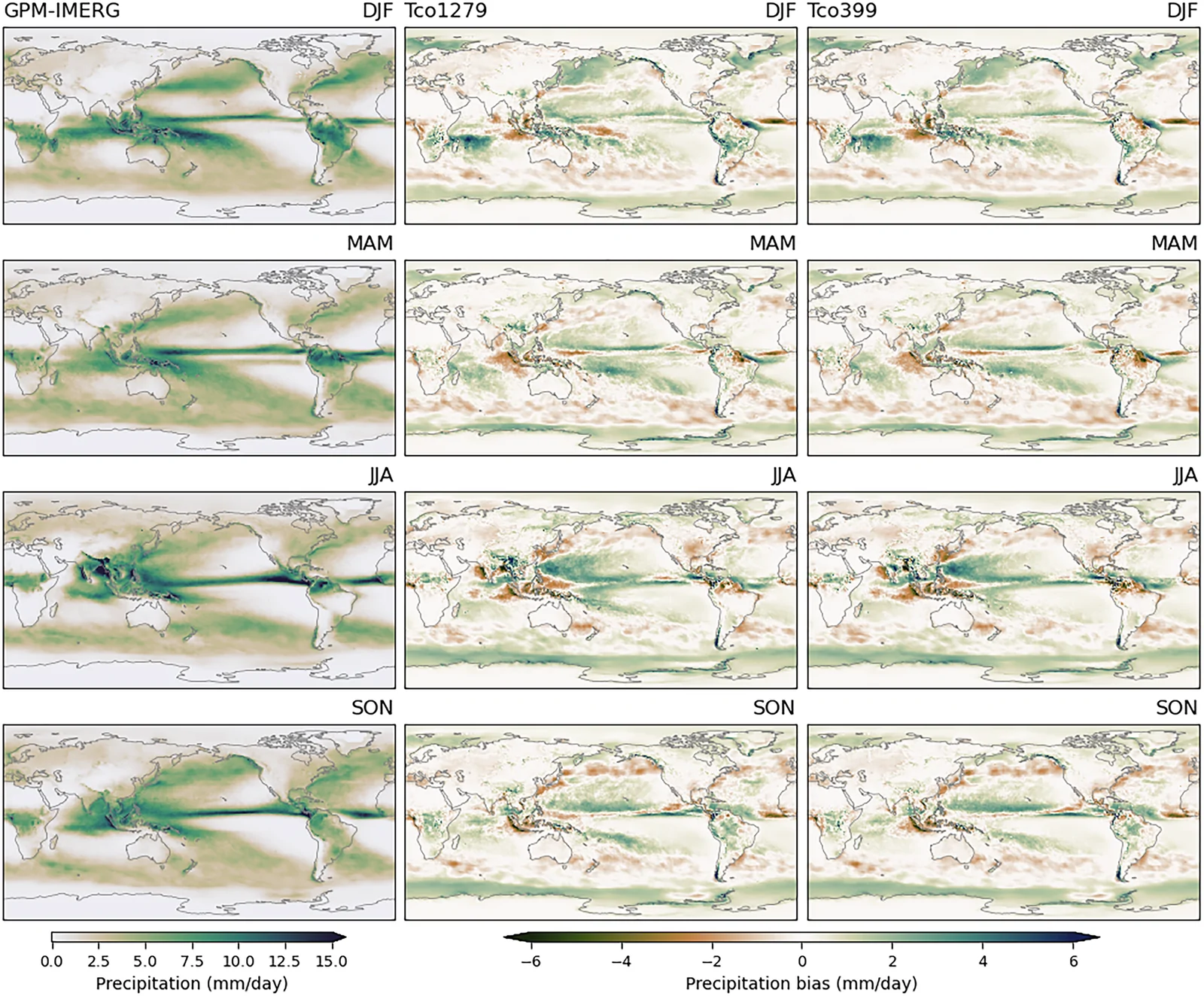
Total precipitation observed climatology from GPM-IMERG (left) for the period 1998-2023 and model biases for (centre) IFS_48R1_Tco1279_EERIE and (right) IFS_48R1_Tco399_EERIE.

An evaluation of near-global mean precipitation is limited by the lack of agreement between the ERA5 reanalysis^24^ and the GPCP^54^ and GPM-IMERG V07B^55^ datasets based on satellite and in-situ observations (Fig. 3c). While IFS simulations overestimate global-average precipitation compared to observations, they are in much closer agreement with ERA5 estimates of global mean precipitation, which likely reflects the similarity of the underlying model used to generate the IFS-based ERA5 global reanalysis. Despite these differences in estimates of near-global mean precipitation, ERA5, GPM-IMERG^55^, and IFS simulations generally show good agreement in the spatial structure of seasonal precipitation climatologies (Figs. 4 and 5).

**Fig. 6:**
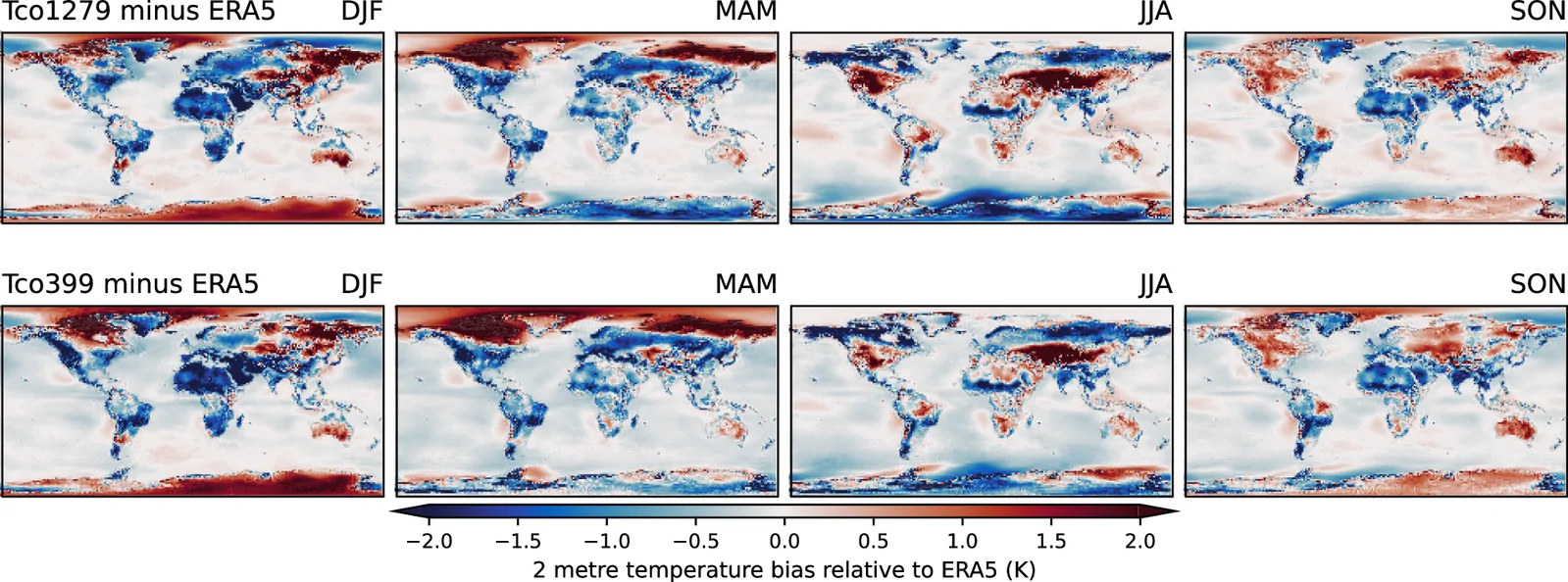
Seasonal mean 2 metre temperature biases relative to ERA5^24^ for (top) IFS_48R1_Tco1279_EERIE and (bottom) IFS_48R1_Tco399_EERIE for the period 1980-2023.

**Fig. 7:**
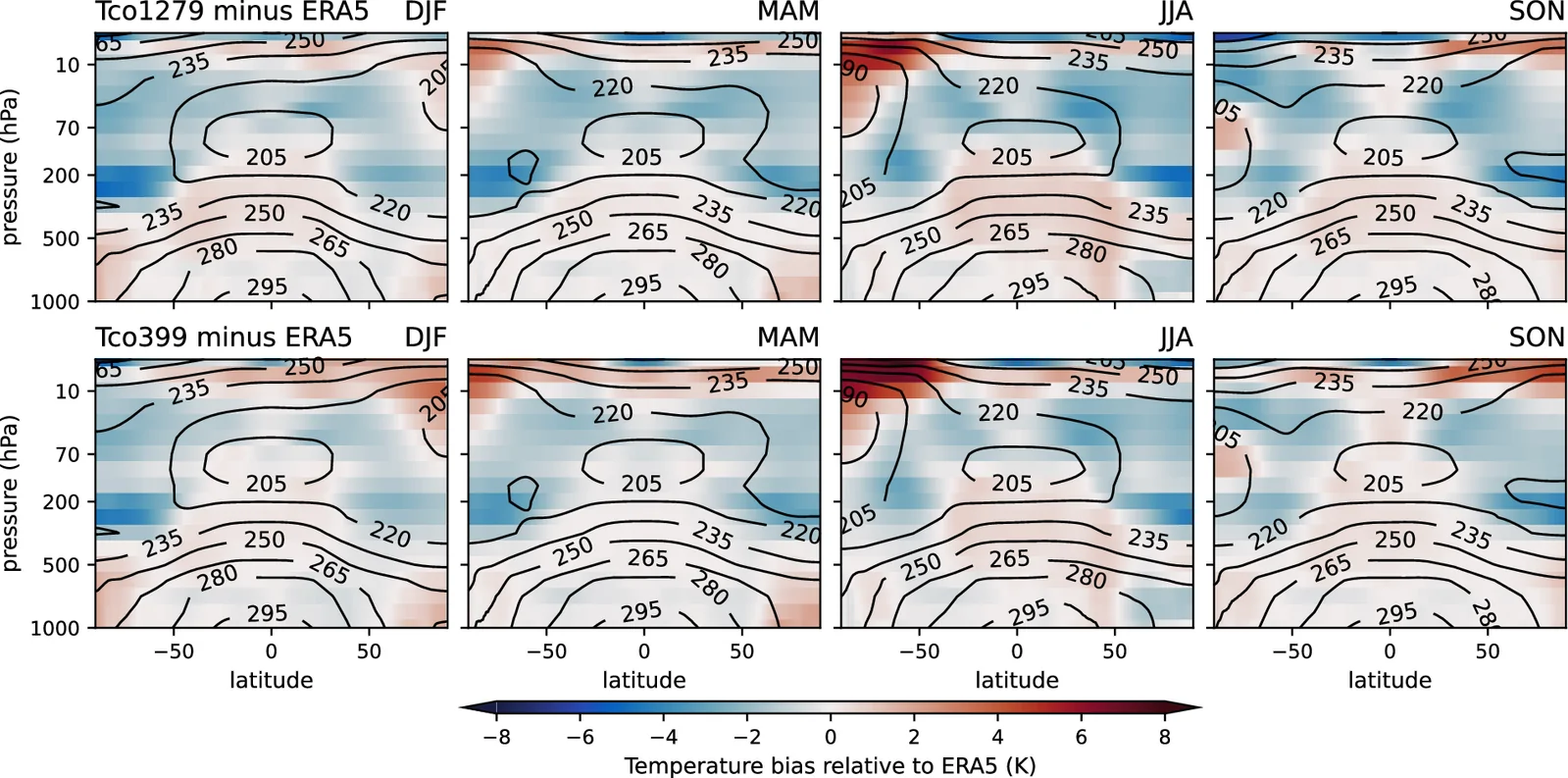
Seasonal zonal mean temperature bias on pressure levels relative to ERA5^24^ for (top) IFS_48R1_Tco1279_EERIE and (bottom) IFS_48R1_Tco399_EERIE for the period 1980-2023. Black contours give ERA5 climatological temperature in K.

**Fig. 8:**
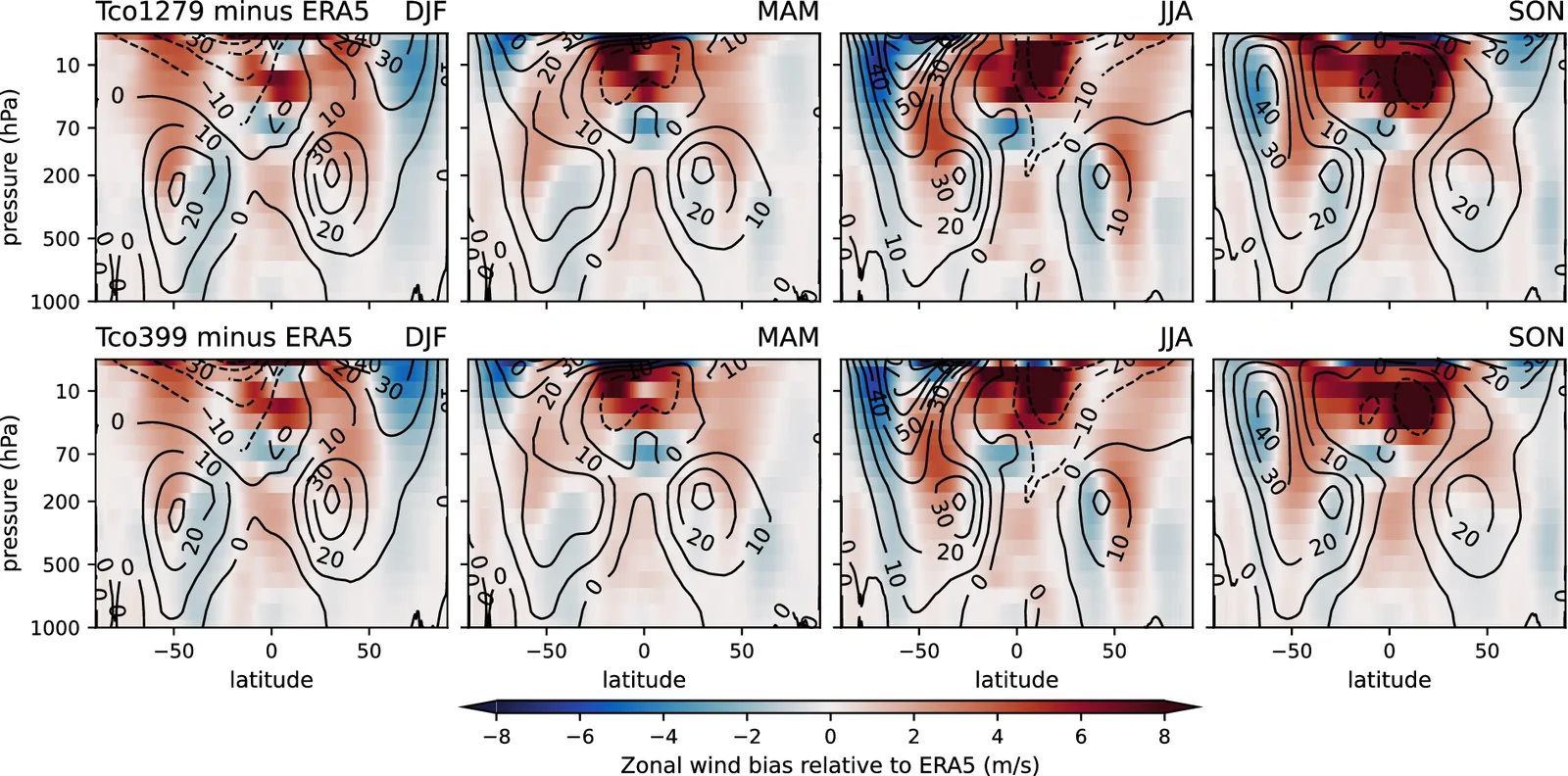
As Fig. 7 but for zonal mean zonal wind on pressure levels.

Seasonal mean 2 metre temperature biases relative to ERA5 (Fig. 6) are generally small over the ocean but can be substantial over land with significant seasonal variations. The spatial patterns and magnitudes of 2 metre temperature biases are very similar in Tco399 and Tco1279 simulations. Seasonal biases in zonal mean temperature and zonal mean zonal wind are shown in Figs. 7 and 8, respectively. Consistent with previous multi-decadal atmosphere-only simulations with an older IFS version^2^, tropospheric temperature biases show limited sensitivity to changes in horizontal resolution. However, we find some evidence for a larger magnitude cold bias in the stratosphere around 50 hPa in the higher resolution Tco1279 simulations. This is likely a consequence of discretization errors in the vertical advection associated with vertically propagating gravity waves, which becomes more problematic at higher horizontal resolutions^56^. However, these errors are substantially reduced compared to previous versions of the IFS used in CMIP6/HighResMIP^2^. These improvements can be attributed to the higher-order vertical interpolation in the semi-lagrangian advection scheme and an increased number of vertical levels used in these simulations, both of which alleviate temperature biases related to the vertical propagation of gravity waves^22,56^.

**Fig. 9:**
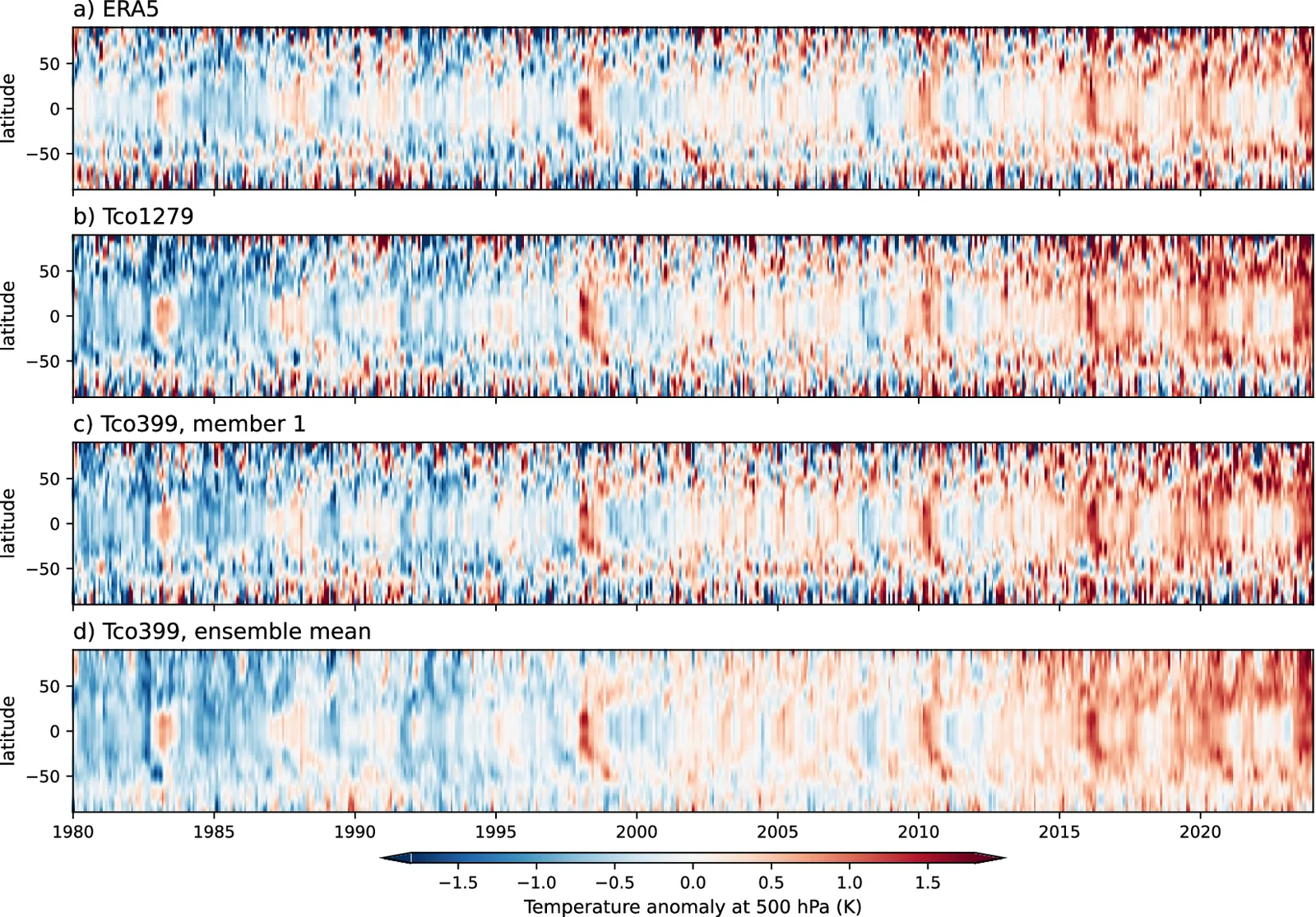
Time-evolution of monthly mean zonal mean 500 hPa temperature anomalies calculated with respect to a 1980–2023 climatology.

IFS atmosphere-only simulations also simulate long-term trends in tropospheric temperature, which are modulated by higher-frequency variations associated with intraseasonal and interannual modes of climate variability (Fig. 9a-c). IFS Tco399 and Tco1279 simulations slightly overestimate the rate of long term warming at 500 hPa compared to ERA5, but accurately simulate the timing and magnitude of tropospheric temperature anomalies associated with major El Niño-Southern Oscillation (ENSO) events. Atmospheric signals attributable to time-varying boundary conditions, such as those related to ENSO SST forcing, are more evident in the IFS Tco399 10-member ensemble mean (Fig. 9d) due to the cancellation of temperature anomalies of arbitrary phase associated with unforced atmospheric weather and climate variability.

Both the Tco1297 and Tco399 simulations display strongly reduced errors compared to coupled CMIP6 models over the 1989–2014 period as evaluated by the CMPItool^57–59^ (not shown). This can be partially explained by the strong SST constraint which prevents model drift but nevertheless underlines the value of these simulations as a useful representation of the evolution of the state of the global atmosphere over the historical period.

## Example application: the local atmospheric response to mid-latitude ocean eddies

One of the goals of the EERIE project, to which these simulations are a contribution, is to understand the local and remote impacts of SST gradients and variability associated with ocean boundary currents and mesoscale ocean eddies on atmospheric weather and climate variability. In this section, we demonstrate that IFS Tco399 and Tco1279 simulations are able to simulate a local atmospheric response to SST anomalies associated with ocean mesoscale eddies. To do this, we follow previous studies^60–62^ and combine observation-based estimates of Southern Ocean eddy locations with an eddy-centric composite analysis of IFS model fields. These composites provide an estimate of the average impact of an ocean eddy on the atmospheric field of interest.

Anticyclonic and cyclonic ocean eddy locations are derived from sea surface height observations^63^ using py-eddy-tracker^64,65^. Based on these locations, composites are constructed by sampling data (observations, model output) relative to the eddy core on a 0.25^°^ × 0.25^°^ latitude-longitude grid, scaled to the individual eddy radius and rotated according the large-scale wind direction (computed over three eddy radii). Each eddy is sampled with 30 points per eddy radius in both the along-wind and across-wind directions. Sensitivity tests with different spatial resampling densities (not shown) indicate that this procedure is adequate to sample the atmospheric response to eddies at these latitudes.

We composite daily mean SST, 10 m wind speed, ocean-atmosphere turbulent (latent + sensible) heat fluxes and total precipitation for the Tco1279 and Tco399 simulations from more than one million of observed cyclonic and anticyclonic eddy locations identified between 25^°^S and 60^°^S during the years 2011–2021. Only every 10th day is sampled, which allows us to cover a longer period in time and avoids sampling individual eddies too often. The same is done for three observational products: SST from ESA CCI v3^34^ (the same as used to force the model), 10 m wind speed from bias-corrected ERA5^66^, and total precipitation from GPM-IMERG^55^.

**Fig. 10:**
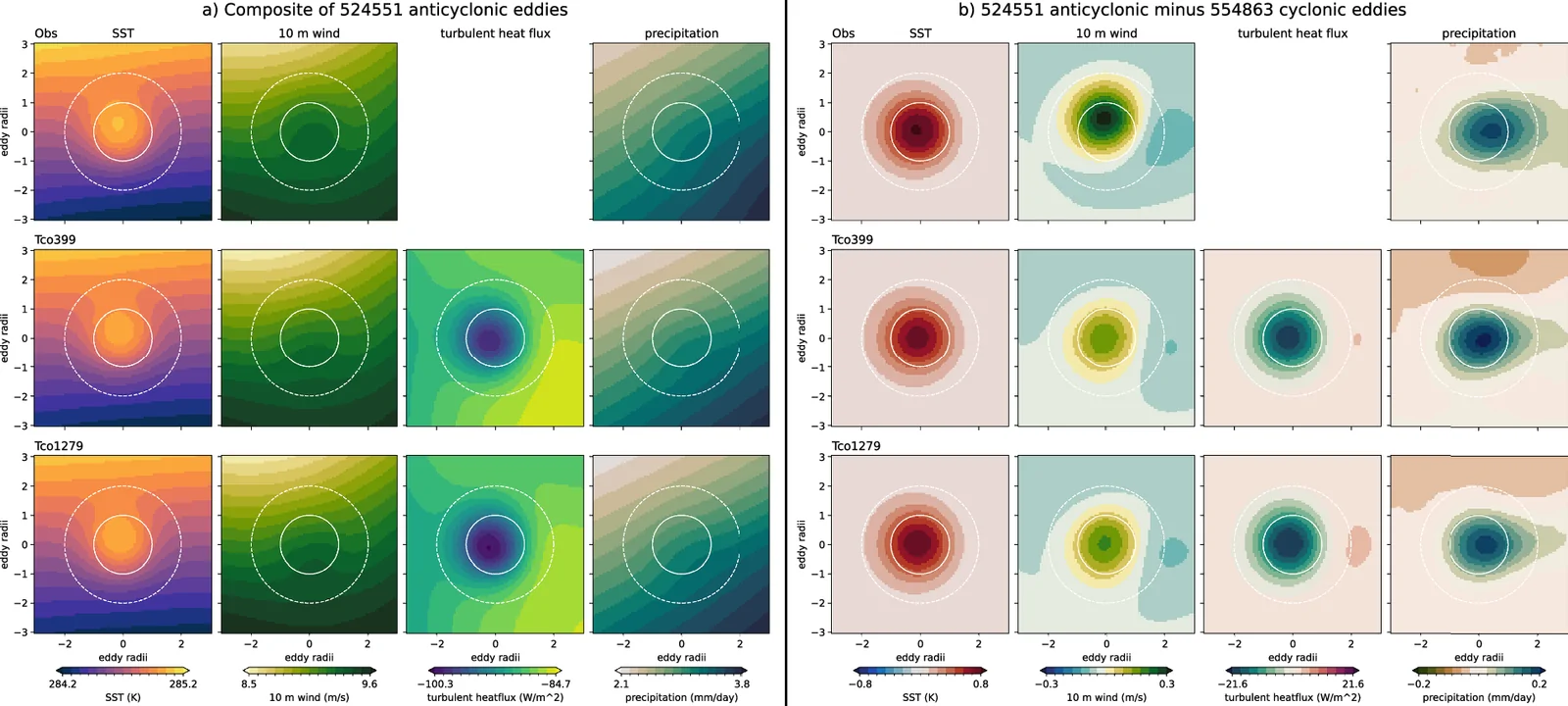
**(a)** Eddy-centric composite means of sea surface temperature (SST), 10 m wind speed, ocean-atmosphere turbulent heat fluxes (positive into the ocean), and total precipitation derived from daily mean data sampled using 524,551 observed anticyclonic eddies locations from the Southern Ocean (25^°^S-60^°^S) between 2010 and 2021. Before calculating composite means, each daily mean snapshot is rotated such that the large scale winds are from left to right and resampled according to the identified eddy radius. Observational composite means are derived using SST from ESA CCI v3^34^, 10 m wind speed from bias-corrected ERA5^66^ and precipitation from GPM-IMERG^55^. IFS composite means are derived from the first member of IFS_48R1_Tco399_EERIE and (bottom) IFS_48R1_Tco1279_EERIE. **(b)** As panel **(a)**, but showing the difference between composites means derived from 524,551 anticyclonic and 554,863 cyclonic eddy locations.

Figure 10 compares the resulting composites for observations and model simulations. Consistent with previous work^60^, our observation-based composites indicate that warm-core anticyclonic eddies in the Southern Ocean are associated with slight increases to 10 m wind speed and precipitation relative to the background environment (Fig. 10a). The impact of ocean eddies on the local marine atmospheric boundary layer is clearer when presented as differences between anticyclonic and cyclonic composite means (Fig. 10b).

The atmosphere-only IFS simulations presented in this study are constrained using the ESA-CCI v3 SST product and thus accurately reproduce the SST structures associated with observed ocean eddy locations (based on sea surface height). Nevertheless, there are some small differences between observed and IFS SST eddy composites as a result of interpolation effects. In general, both Tco399 and Tco1279 simulations accurately reproduce the main features of the observed composite mean response of the atmospheric boundary layer to Southern Ocean eddies (Figs. 10a-b). Specifically, warm SST anomalies are associated with an anomalous turbulent heat flux from the ocean to atmosphere, which stimulates positive anomalies in 10m wind speed and total precipitation that have a limited sensitivity to IFS horizontal resolution. Although IFS-based precipitation composites mean anomalies are similar (Tco1279) or slightly stronger (Tco399) than observation-based estimates, we note that the IFS-based 10m wind anomalies associated with ocean eddies are weaker than observed. This could be a consequence of missing coupled feedbacks that are not represented in these experiments. Nevertheless, we conclude that both EERIE IFS configurations have sufficient horizontal resolution to capture a local atmospheric response to SST anomalies associated with ocean mesoscale eddies.

## Data Availability

The EERIE dataset is freely available in the ECMWF Meteorological Archival and Retrieval System (MARS), with the Tco1279 simulation denoted by experiment ID 0001 (https://apps.ecmwf.int/ifs-experiments/ed/0001/) and the Tco399 10-member ensemble denoted by experiment ID 0002 (https://apps.ecmwf.int/ifs-experiments/ed/0002/). The pages include sample MARS requests to retrieve the data via the ECMWF Web API. The data hosted at DKRZ can be explored via the EERIE cloud starting from https://discover.dkrz.de/external/stac2.cloud.dkrz.de/fastapi/collections/eerie.

## Code availability

The IFS source code is available subject to a license agreement with ECMWF. ECMWF member-state weather services and their approved partners will be granted access. The IFS code without modules for data assimilation is also available for educational and academic purposes as part of the OpenIFS project^67^.
